# Prone Positioning in Awake, Nonintubated Patients With COVID-19 Hypoxemic Respiratory Failure

**DOI:** 10.1001/jamainternmed.2020.3030

**Published:** 2020-06-17

**Authors:** Alison E. Thompson, Benjamin L. Ranard, Ying Wei, Sanja Jelic

**Affiliations:** 1 Division of Pulmonary, Allergy, and Critical Care Medicine, Columbia University Vagelos College of Physicians and Surgeons, New York, New York; 2 Division of Biostatistics, Columbia University Vagelos College of Physicians and Surgeons, New York, New York

## Abstract

This cohort study investigates whether the prone position is associated with improved oxygenation and decreased risk for intubation in spontaneously breathing patients with severe COVID-19 hypoxemic respiratory failure.

Critically ill patients with coronavirus disease 2019 (COVID-19) severely strained intensive care resources in New York City in April 2020.^1^ The prone position improves oxygenation in intubated patients with acute respiratory distress syndrome.^2,3^ We investigated whether the prone position is associated with improved oxygenation and decreased risk for intubation in spontaneously breathing patients with severe COVID-19 hypoxemic respiratory failure.^4,5,6^

## Methods

We screened consecutive patients admitted to the Columbia University step-down unit (intermediate care unit) between April 6 and April 14, 2020 (N = 88). Inclusion criteria were laboratory-confirmed COVID-19 with severe hypoxemic respiratory failure defined as respiratory rate of 30 breaths/min or greater and oxyhemoglobin saturation (Spo_2_) of 93% or less while receiving supplemental oxygen 6 L/min via nasal cannula and 15 L/min via nonrebreather face mask. A confirmed case of COVID-19 was defined by a positive result on a reverse transcriptase–polymerase chain reaction assay of a specimen collected on a nasopharyngeal swab. Exclusion criteria were altered mental status with inability to turn in bed without assistance (n = 13), extreme respiratory distress requiring immediate intubation (n = 23), or oxygen requirements less than those specified in the inclusion criteria (n = 23). We asked eligible patients (n = 29) to lie on their stomach for as long as tolerated up to 24 hours daily. They could use a pillow placed under the hips/pelvis if desired and rest in the lateral decubitus or supine position followed by repeat prone positioning. Do-not-resuscitate status did not affect the decision to initiate or continue the use of the prone position. The Columbia University institutional review board approved the study and waived the need for informed consent from the participants, as we analyzed deidentified data collected from electronic medical records. The primary outcome was change in Spo_2_ before and 1 hour after initiation of the prone position. We report the median change in Spo_2_ with 95% CIs. We used the Wilcoxon test for analysis of change in Spo_2_. We assessed the mean risk difference in intubation rates for patients with Spo_2_ of 95% or greater vs Spo_2_ less than 95% 1 hour after initiation of the prone position. We assessed intubation rates across demographic and other clinical factors with RStudio, version 1.2.5019 (RStudio).

## Results

Among 29 eligible patients, 25 had at least 1 awake session of the prone position lasting longer than 1 hour; 4 refused the prone position and were intubated immediately. One hour after initiation of the prone position, Spo_2_ increased compared with baseline (Figure). The range of improvement in Spo_2_ was 1% to 34% (median [SE], 7% [1.2%]; 95% CI, 4.6%-9.4%). In all patients, the levels of supplemental oxygen were unchanged during the first hour of the prone position. One hour after initiation of the prone position, 19 patients had Spo_2_ of 95% or greater; subsequently, 7 (37%) required intubation. Among 6 patients whose Spo_2_ remained less than 95% 1 hour after initiation of the prone position, 5 (83%) were intubated. The mean difference in the intubation rate among patients with Spo_2_ of 95% or greater vs Spo_2_ less than 95% 1 hour after initiation of the prone position was 46% (95% CI, 10%-88%). The Table shows other patient characteristics, none of which were associated with the need for intubation. Among 12 patients who required intubation, 3 died subsequently in the intensive care unit. Among 13 patients who did not require intubation, 9 recovered and were discharged from the hospital, 2 were transferred to the medical ward, and 2 remained in the step-down unit at the time data were censored on May 25, 2020.

**Figure.**
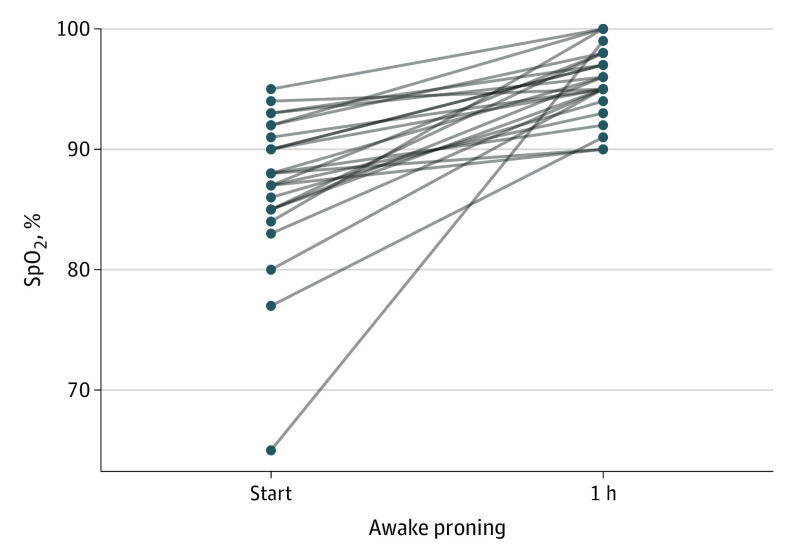
Oxyhemoglobin Saturation (Spo_2_) 1 Hour After Initiation of the Prone Position in Awake, Nonintubated Patients With COVID-19 Spo_2_ before and 1 h after initiation of the prone position in awake, nonintubated patients with COVID-19 severe hypoxemic respiratory failure (n = 25).

**Table.** Bivariate Analysis of Patient Characteristics and Their Association With Intubation After Use of the Prone Position in the 25 Awake, Nonintubated Patients With COVID-19

Characteristic	No. (%)		Intubation rate difference, % (95% CI)^a^
	Not intubated (n = 13)	Intubated (n = 12)	
Age, median (range), y	67.0 (45.0 to 71.0)	66.0 (53.0 to 87.0)	4 (−35 to 43)
Sex (female)	3 (23)	4 (33)	7 (−36 to 50)
Body mass index, median (range)^b^	29.0 (21.0 to 47.0)	27.5 (22.0 to 33.0)	−4 (−43 to 35)
Hypertension	7 (54)	5 (42)	12 (−26 to 51)
Diabetes	5 (39)	5 (42)	−3 (−43 to 36)
Hyperlipidemia	1 (8)	2 (17)	−21 (−78 to 36)
Coronary artery disease	1 (8)	1 (8)	−2 (−74 to 70)
Chronic lung disease^c^	2 (15)	2 (17)	−2 (−74 to 70)
Chronic kidney disease	1 (8)	0	NA
Symptom onset to prone position, median (range), d	12.0 (6.0 to 24.0)	12.0 (4.0 to 19.0)	−20 (−59 to 19)
Days from admission to prone position, median (range)	3.0 (1.0 to 12.0)	3.5 (1.0 to 7.0)	−20 (−59 to 19)
Duration of prone position on day 1, median (range), h	4.0 (1.0 to 24.0)	6.0 (1.0 to 24.0)	−35 (−72 to 0)
Days for use of the prone position, median (range)	2.0 (1.0 to 5.0)	2.0 (1.0 to 3.0)	26 (−13 to 67)

Abbreviation: NA, not applicable.

^a^ For a binary risk factor *x*, the intubation risk difference is defined by Δ = [*intubation rate* ׀ *x = yes*] − [*intubation rate* ׀ *x = no*]. When *x* is a continuous risk factor, the intubation risk difference is defined by Δ *=* [*intubation rate* ׀ *x* ≥ *median*] − [*intubation rate* ׀ *x* < *median*]. The 95% CI of Δ is constructed by Δ ± SE_Δ_ where SE_Δ_ is the standard error of Δ. None of the differences were significant.

^b^ Calculated as weight in kilograms divided by height in meters squared.

^c^ Chronic lung disease includes asthma, chronic obstructive pulmonary disease, and interstitial lung disease.

## Discussion

In this small single-center cohort study, we found that the use of the prone position for awake, spontaneously breathing patients with COVID-19 severe hypoxemic respiratory failure was associated with improved oxygenation. In addition, patients with an Spo_2_ of 95% or greater after 1 hour of the prone position was associated with a lower rate of intubation. Limitations of our study are the lack of control group and a small sample size. Randomized clinical trials are needed to establish whether improved oxygenation after use of the prone position in awake, nonintubated patients improves survival.
